# Enhanced ROS scavenging and sugar accumulation contribute to drought tolerance of naturally occurring autotetraploids in *Poncirus trifoliata*

**DOI:** 10.1111/pbi.13064

**Published:** 2019-01-10

**Authors:** Tonglu Wei, Yue Wang, Zongzhou Xie, Dayong Guo, Chuanwu Chen, Qijun Fan, Xiaodong Deng, Ji‐Hong Liu

**Affiliations:** ^1^ Key Laboratory of Horticultural Plant Biology (MOE) College of Horticulture and Forestry Sciences Huazhong Agricultural University Wuhan China; ^2^ Guangxi Key Laboratory of Citrus Biology Guangxi Academy of Specialty Crops Guilin China

**Keywords:** *Poncirus trifoliata*, autotetrploid, drought tolerance, RNA‐seq, tetraploid, ROS, sugar metabolism

## Abstract

Tetraploids have been reported to exhibit increased stress tolerance, but the underlying molecular and physiological mechanisms remain poorly understood. In this study, autotetraploid plants were identified by screening natural seedlings of trifoliate orange (*Poncirus trifoliata*). The tetraploids exhibited different morphology and displayed significantly enhanced drought and dehydration tolerance in comparison with the diploid progenitor. Transcriptome analysis indicated that a number of stress‐responsive genes and pathways were differentially influenced and enriched in the tetraploids, in particular those coding for enzymes related to antioxidant process and sugar metabolism. Transcript levels and activities of antioxidant enzymes (peroxidase and superoxide dismutase) and sucrose‐hydrolysing enzyme (vacuolar invertase) were increased in the tetraploids upon exposure to the drought, concomitant with greater levels of glucose but lower level of reactive oxygen species (ROS). These data indicate that the tetraploids might undergo extensive transcriptome reprogramming of genes involved in ROS scavenging and sugar metabolism, which contributes, synergistically or independently, to the enhanced stress tolerance of the tetraploid. Our results reveal that the tetraploids take priority over the diploid for stress tolerance by maintaining a more robust system of ROS detoxification and osmotic adjustment via elevating antioxidant capacity and sugar accumulation in comparison with the diploid counterpart.

## Introduction

Whole‐genome duplication (WGD) or polyploidy occurs extensively in wild plant species and most angiosperms have undergone one or more WGD events during their evolution (Jiao *et al*., 2011). Many important crops, such as wheat, canola, potato, sugarcane and cotton, are polyploids (Soltis and Soltis, 2016). Polyploids generally arise by autopolyploidy and allopolyploidy, in which the chromosomes are derived from a single species or from different species, leading to generation of autopolyploids and allopolyploids respectively. Allopolyploids originate primarily from interspecific hybridization and are composed of distinguishable subgenomes, whereas autotetraploids result from somatic chromosome doubling or intraspecific hybridization (Allario *et al*., 2013; Stupar *et al*., 2007).

It has been well‐known that polyploids exhibit unique characteristics relative to their diploid ancestors due to a greater abundance of genetic opportunities. Previous studies have demonstrated that a range of genetic alterations are associated with allopolyploidy than with autopolyploidy, mainly due to the complexity of the inherited genetic background of allopolyploids (Yang *et al*., 2014; Zhang *et al*., 2015c). The phenotype of allopolyploids is strongly dependent on the composition of different subgenomes and commonly reflects evolutionary and domestication influences (Comai, 2005). In contrast, autopolyploids exhibit negligibly dramatic changes in genetic potential, at least over a short time‐scale; the autopolyploids have been relatively less investigated. However, increasing attention has been invested to autopolyploids due to their profound effects on plant morphology and physiology relative to their diploid progenitors. Such alterations may include larger organ structure (Dudits *et al*., 2016), slower growth (Allario *et al*., 2011), improved fruit yield (Hussain *et al*., 2012) and different anatomical features (Allario *et al*., 2011). In addition, accumulating evidence demonstrate that autotetraploids exhibit dramatically enhanced tolerance to a variety of abiotic stresses, including cold (Oustric *et al*., 2017), drought (Allario *et al*., 2013), salt (Liu and Sun, 2017; Ruiz *et al*., 2016b; Tu *et al*., 2014), heat (Zhang *et al*., 2010), chromium toxicity (Balal *et al*., 2017) and boron excess (Ruiz *et al*., 2016a). These observations provide the impetus for additional research to understand the underlying basis for many of these desirable performance and traits resulting from autopolyploidy.

Plants are constantly challenged by a myriad of abiotic stresses during their life span. On the other hand, plants have also evolved a range of defensive approaches to cope with the adverse stimuli. So far, tremendous progresses have been achieved in understanding the mechanism by which plants adapt to and cope with the single or multiple stresses (Zhu, 2016). It is now widely accepted that plants combat with the harsh environmental cues by mobilizing transcriptional alteration of a vast number of stress‐responsive genes, leading to activation of critical enzymes or accumulation of various metabolites that play direct or indirect roles in preventing plant cells from stress‐associated damages (Liu *et al*., 2014). However, it has to be mentioned that these theories or viewpoints are predominantly garnered by pairwise comparison of diploids, and there is a great paucity of knowledge concerning the molecular events associated with stress response of the autotetraploids.

It is generally envisaged that comparisons of autopolyploids with their diploid parents can provide greater insight into mechanisms underlying increased stress tolerance of the former. Despite the importance of this topic, relevant advancements in our understanding have been limited and are mainly confined to physiological and biochemical response (Balal *et al*., 2017; Liu *et al*., 2011; Meng *et al*., 2016; Podda *et al*., 2013; Tu *et al*., 2014). While the broad advantages of historical or evolutionary autopolyploidy on environmental adaption are commonly recognized (Comai, 2005; Fasano *et al*., 2016; Parisod *et al*., 2010), the improved stress tolerance of neopolyploids and the underlying molecular mechanisms have not been explicitly investigated. With the advent of high throughput sequencing, differences in transcriptome and miRNAome profiles between diploids and polyploids in the presence of a given stressor are investigated (Allario *et al*., 2011; Cao *et al*., 2017; Fan *et al*., 2016; Liu and Sun, 2017; Stupar *et al*., 2007). These studies demonstrated that a limited number of the transcribed genes are differentially expressed, implying that polyploidization may cause subtle changes in the expression of specific genes responsible for stress tolerance (Allario *et al*., 2013; Tan *et al*., 2015). Very recently, Zhang *et al*. (2015a) reported that expression of microRNAs and stress‐related genes was altered by DNA methylation in polyploid rice. Collectively, however, even though the increased stress tolerance of polyploids is well recognized, many aspects of the underlying bases for the increased tolerance are still unknown. In particular, it is worth mentioning that very few studies have combined both physiological and molecular analyses to elucidate the mechanisms.

Diploid trifoliate orange (*Poncirus trifoliata* (L.) Raf.) is widely used as a rootstock for citrus due to its favourable adaptation to a variety of environmental conditions, such as cold hardiness and tolerance to several diseases (Boava *et al*., 2011; Gong and Liu, 2013). However, trifoliate orange is susceptible to drought (Gong *et al*., 2015), so improvement of drought tolerance is a major goal so that citrus trees grafted onto trifoliate orange can be more tolerant in the case of water deficiency. Since polyploids exhibit desirable stress tolerance, it is proposed that tetraploid trifoliate orange may hold great potential for improving drought resistance. Previously tetraploids of trifoliate orange were reported to exhibited enhanced salt stress (Mouhaya *et al*., 2010; Saleh *et al*., 2008), but is remains to be determined whether trifoliate orange tetraploids can display improved drought tolerance. In this study, we successfully obtained autotetraploids of trifoliate orange and demonstrated that they were more tolerant to drought and dehydration stress than the diploids. Global transcriptome analysis based on RNA‐sequencing (RNA‐seq) showed that the tetraploids exhibited different transcriptome profiling in comparison with the diploid, in which genes involved in antioxidant system and sugar metabolism were uniquely and specifically enriched in the tetraploid. Further studies indicate that that autotetraploid trifoliate oranges exhibited enhanced capacity for ROS scavenging and osmotic adjustment via activating antioxidant and sucrose‐hydrolysing genes, which may contribute to imparting the stress tolerance.

## Results

### Screening and molecular identification of tetraploid plants

**Figure 1:**
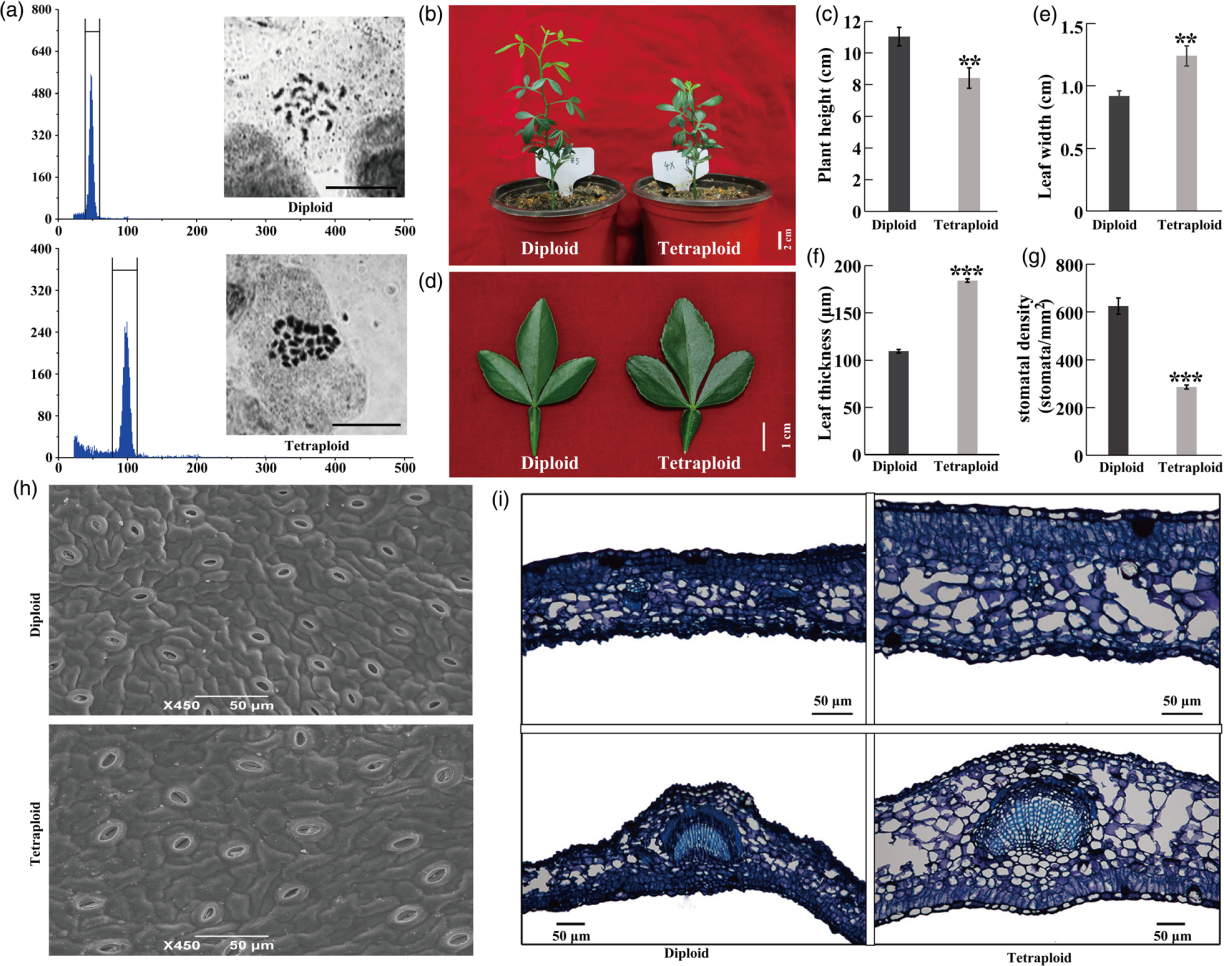
Morphological and microscopic comparison between tetraploid and diploid. (a) Ploidy level of diploid and tetraploid plants, as determined by FCM (flow cytometry) and chromosome counting. Upper: diploid, bottom: tetraploid. Bar = 10 μm. (b–f) Plant morphology (b), plant height (c, *n* = 12), leaf morphology (d), leaf width (e, *n* = 20) and leaf thickness (f, *n* = 20) in 4‐month‐old diploid and tetraploids. (g) Stomatal density (*n* = 20). (h) Scanning electronic microscopic observation of stomata in diploid and tetraploid leaves. (i) Microscopic observation using cross sections of diploid and tetraploid leaves. Error bars indicate SE. Asterisks indicate significant differences between diploid and tetraploid (** *P *<* *0.01; *** *P *<* *0.001).

It has been previously suggested that tetraploids frequently occur in apomictic genotypes of citrus and related genera (Aleza *et al*., 2011; Tan *et al*., 2015). This implies that tetraploids can be identified in natural seedlings derived from apomictic plants. Based on this rationale, a preliminary screening of 1130 plants germinating from seeds of trifoliate orange was conducted, and a total of 31 plants were assumed to be potential tetraploids based on the unique morphological characteristics associated with tetraploids, such as thicker and greener leaves and smaller plant height. The ploidy level of the 31 putative tetraploids was determined using both flow cytometry (FCM) and chromosome count. FCM indicated that the fluorescence intensity of the tetraploid cells peaked at about 100, a value that was twofold of that in the diploid control. The analysis of chromosome number in root tips indicated that the diploids contained 18 chromosomes, whereas 36 chromosomes were detected in the tetraploids (Figure 1a). As a result, FCM and chromosome count indicated that 22 of the 31 putative tetraploid plants were indeed tetraploids.

Genome re‐sequencing of a representative tetraploid line and the diploid progenitor was performed in order to further characterize the genomic composition and genetic fidelity of the tetraploid (Table S1). Since genome sequence of trifoliate orange is not currently available, the pummelo genome was used as a reference to identify SNPs in the diploid and tetraploid. Genome sequence alignment showed that the diploid and tetraploid plants shared a total of 3 189 570 SNPs, representing a 94.2% sequence identity between each other (Figure S1a). A phylogenetic analysis based on the SNPs identified in the diploid and tetraploid trifoliate orange, three pummelo genotypes and three mandarin genotypes was also conducted. Results indicated that the tetraploid was most closely related to the diploid trifoliate orange (Figure S1b). Collectively, the data indicate that the tetraploids are autotetraploids originating from a natural doubling of the diploid genome.

### Morphological and microscopic comparisons of tetraploids and diploids

Under normal growing conditions in the greenhouse, the tetraploid plants exhibited a noticeable difference in plant morphology relative to their diploid progenitors. The tetraploid plants grew more slowly than the diploids, resulting in significantly shorter plant height (Figure 1b, c). The leaves of the tetraploids were prominently larger and thicker than those of the diploids (Figure 1d–f). In addition, the tetraploids had a dramatically smaller stomatal density relative to the diploids (Figure 1g, h). Microscopic observation revealed conspicuous anatomical differences between the tetraploids and diploids, as manifested by larger cells and thicker epidermis and palisade tissues in the tetraploids (Figure 1i). These observations clearly indicated that the tetraploids were morphologically and anatomically different from their diploid progenitor.

### Tetraploid plants exhibit enhanced drought and dehydration tolerance

**Figure 2:**
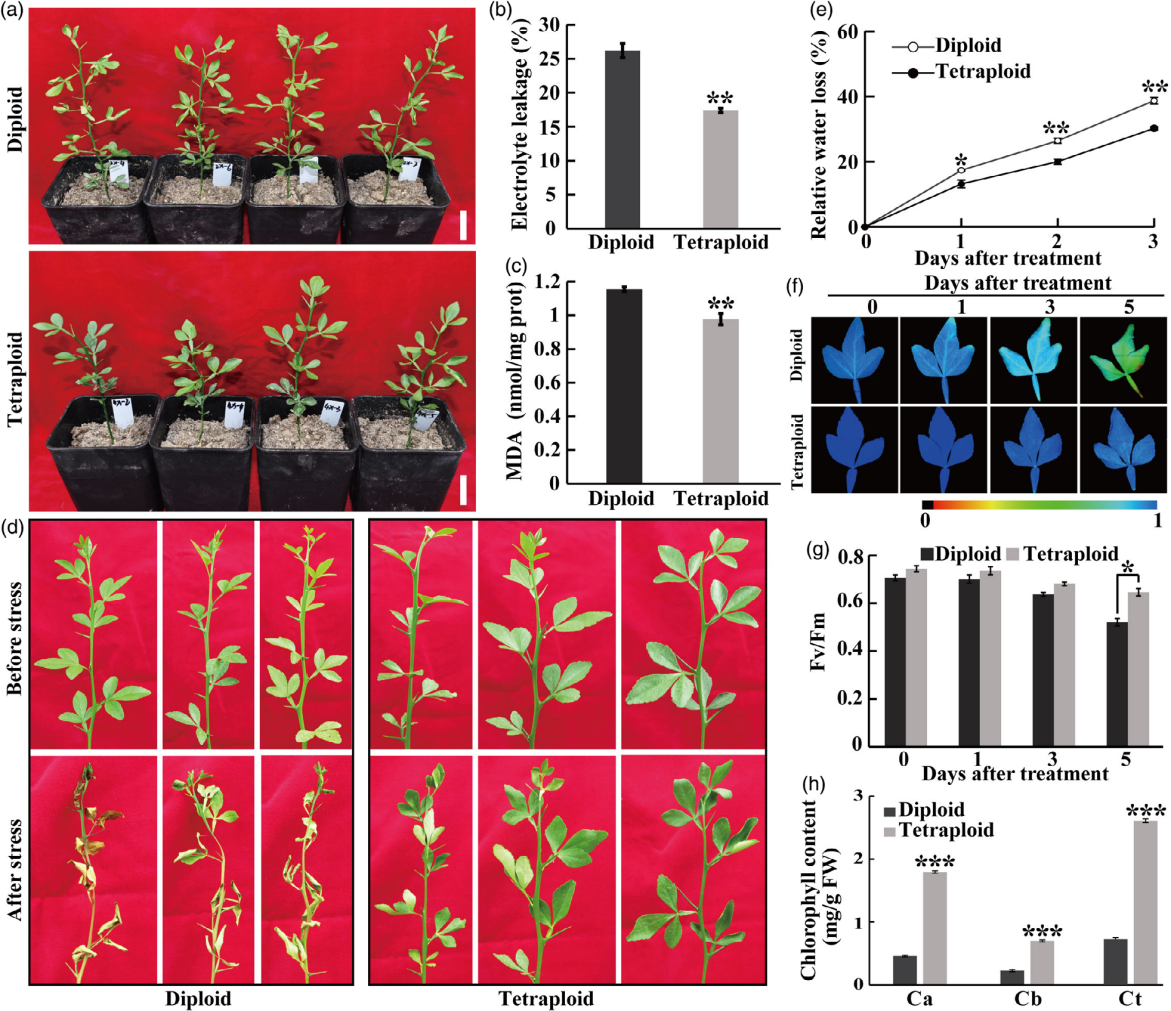
The tetraploid trifoliate orange plants displayed enhanced drought and dehydration tolerance relative to the diploids. (a–c) Plant phenotype (a), electrolyte leakage (b) and MDA content (c) of diploid and tetraploids after being subjected to a 28‐day drought treatment. Bar = 3 cm. (d) Phenotype of fresh shoots detached from diploid and tetraploid plants before and after 3‐day dehydration stress. (e) Relative fresh water loss rate in diploid and tetraploid leaves. (f–g) Chlorophyll fluorescence image (f) and the corresponding *F*_v_/*F*_m_ ratios (g) of diploid and tetraploid over the course of a 5‐day dehydration treatment. The colour barcode is shown below the images. (h) Chlorophyll content (a, b and total) in diploid and tetraploid after the 3‐day dehydration stress. Error bars indicate SE (*n* = 3). Asterisks indicate significant differences between diploid and tetraploid (* *P *<* *0.05; ** *P *<* *0.01; *** *P *<* *0.001).

Trifoliate orange is known to be sensitive to drought stress. Therefore, it was of interest to determine whether the tetraploids exhibited improved drought tolerance relative to the diploids. Therefore, 4‐month‐old diploid and tetraploid plants were subjected to drought stress by withholding watering for 28 day. At the end of drought treatment, leaf wilting was more serious in the diploid plants than in the tetraploids (Figure 2a). EL and MDA levels, two major indicators of membrane damage, were measured after the drought stress, which indicated that the levels of the two parameters were significantly lower in the tetraploids when compared with those of the diploids (Figure 2b, c).

Detached shoots and leaves were subjected to dehydration treatment for three or 5 days. As can be seen in Figure 2d, the diploid shoots exhibited a greater level of leaf wilting and yellowing in comparison with the tetraploid counterparts. In addition, fresh water loss was monitored in the diploid and tetraploid leaves exposed to dehydrative stress; it is noticeable that the diploid leaves lost more water than the tetraploids over the whole course (Figure 2e). Chlorophyll fluorescence imaging demonstrated that the leaves of tetraploids maintained a higher level of chlorophyll fluorescence relative to the diploid (Figure 2f). Consistently, *F*_v_/*F*_m_ ratios of the two populations were progressively reduced by dehydration in both genotypes, however, the tetraploids displayed higher *F*_v_/*F*_m_ ratios than the diploids, with the difference being significant at the last time point (Figure 2g). As a consequence, chlorophyll contents were significantly higher in the tetraploids than in the diploids (Figure 2h). Collectively, these results indicated that the tetraploid plants were more tolerant to drought and dehydration stress than the diploids.

### The global transcriptome is altered in the tetraploids of trifoliate orange

Global transcriptome based on RNA‐seq was analysed so as to further understand the molecular mechanisms underlying the enhanced drought tolerance of the tetraploid trifoliate orange. To this end, the transcriptome profiles of diploid and tetraploid plants under well‐watered conditions (designated 2C and 4C for diploid and tetraploid respectively) and under drought stress (2D and 4D for diploid and tetraploid respectively) were compared. A total of 188 731 771 clean reads were obtained, accounting for 99.9% of the total reads and representing a total of 8 255 637 683 bp. The clean reads were mapped to the sweet orange reference genome, leading to an 88.8% mapping ratio, comprised of 59.0% single matches and 29.8% multiple gene matches (Table S2).

**Figure 3:**
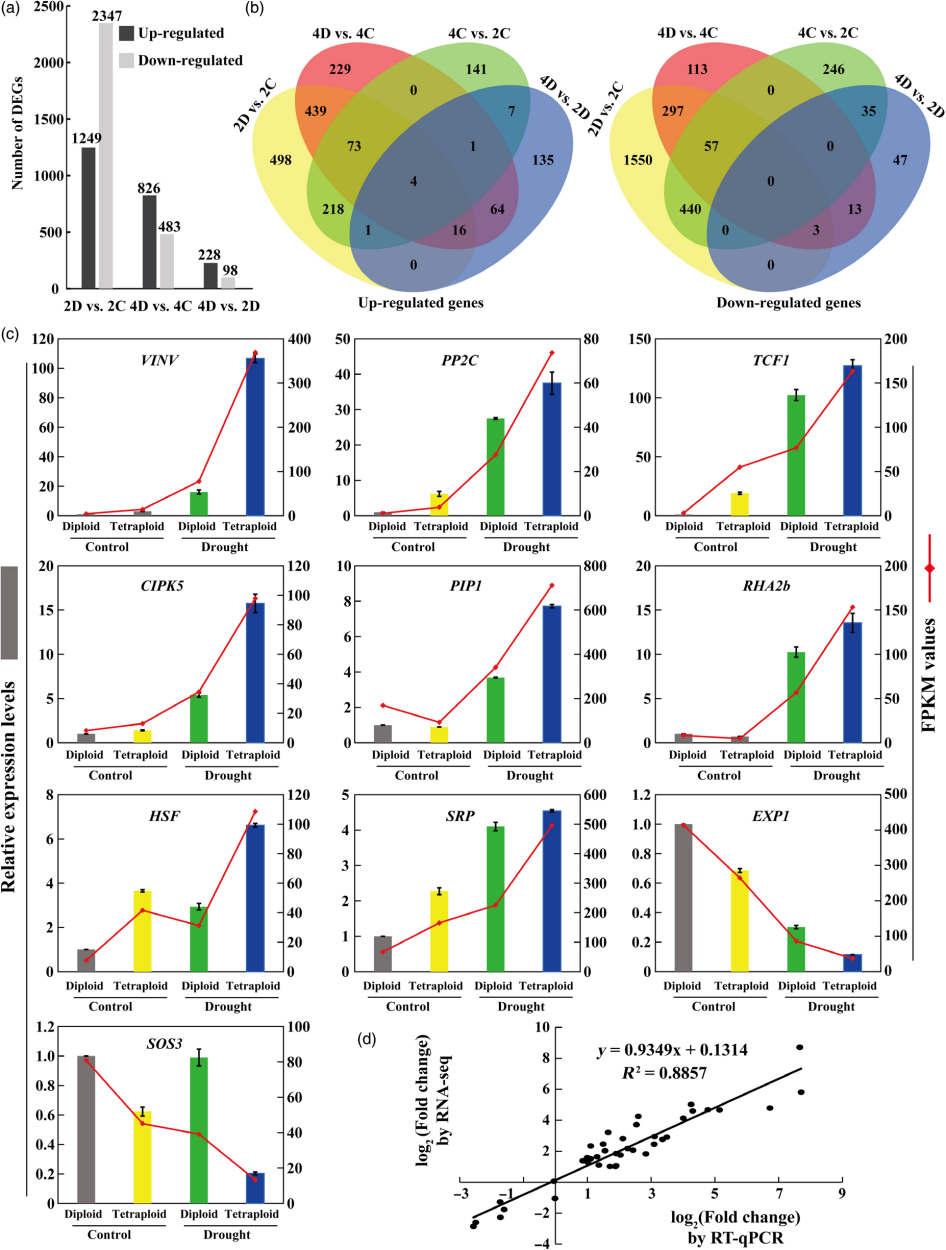
Differentially expressed genes (DEGs) in the diploid and tetraploid plants under control and drought conditions. (a) Number of up‐regulated and down‐regulated genes in the diploid and tetraploid exposed to drought vs. control conditions (2D vs. 2C, 4D vs. 4C) and in tetraploid vs. diploid under drought condition (4D vs. 2D), as revealed by RNA‐seq. (b) Venn diagrams analysis of the common and specific up‐regulated and down‐regulated genes unique and shared between the different pairwise comparisons. (c) Transcript levels of ten selected differentially expressed genes (DEGs), as revealed by RT‐qPCR (bars) and RNA‐seq (red lines). RT‐qPCR data are means ± SE (*n* = 3). *VINV*: vacuolar invertase; *PP2C*: protein phosphatase *2C; TCF1: RCC1* family protein, Tolerant to Chilling and Freezing 1; *CIPK5*: CBL‐interacting serine/threonine‐protein kinase; *PIP1*: plasma membrane intrinsic proteins, aquaporin; *RHA2b*: RING‐H2 E3 ligase; *HSP*: heat shock protein; *SRP*: stress‐related protein; *EXP1*: expansin; *SOS3*: salt overly sensitive 3. (d) Correlation of expression analysis by RNA‐seq (*Y*‐axis) and RT‐qPCR (*X*‐axis).

Transcriptomic differences between the diploid and tetraploid were determined by performing pairwise comparisons (2D vs. 2C, 4D vs. 4C, 4C vs. 2C and 4D vs. 2D) of gene expression levels using the aligned reads so as to identify DEGs in each pair (Tables S3‐S6). As shown in Figure 3a, a total of 3596 DEGs, 1249 up‐regulated and 2347 down‐regulated, were identified in 2D vs. 2C. In contrast, a total of 1309 DEGs were identified 4D vs. 4C, among which 826 were up‐regulated and 483 were down‐regulated by drought treatment, implying that the number of DEGs in response to drought stress was smaller in the tetraploid than in the diploid. In addition, a total of 228 up‐regulated DEGs and 98 down‐regulated DEGs were annotated in 4D vs. 2D. Further analysis indicated that only four genes were commonly up‐regulated in the four sets of pairwise comparisons, whereas no common genes were observed in the down‐regulated ones. In addition, 16 up‐regulated and three down‐regulated genes were overlapped in three pairwise comparisons, 2D vs. 2C, 4D vs. 4C and 4D vs. 2D (Figure 3b). It was reasoned that these 23 DEGs might play key roles in modulating drought tolerance of the tetraploids. The 23 genes included 10 genes with known functions in abiotic stress response, such as *VINV*,* PP2C*,* TCF1*,* CIPK5*,* PIP1*,* RHA2b*,* HSF*,* SRP*,* EXP1* and *SOS3*. Expression patterns of these 10 genes, evaluated by RT‐qPCR, were largely in agreement with the RNA‐seq data, as reflected by a high correlation coefficient (*R* ^2^ = 0.8857) between the two methods (Figure 3c, d).

### GO annotation and KEGG pathway analysis of the DEGs

**Figure 4:**
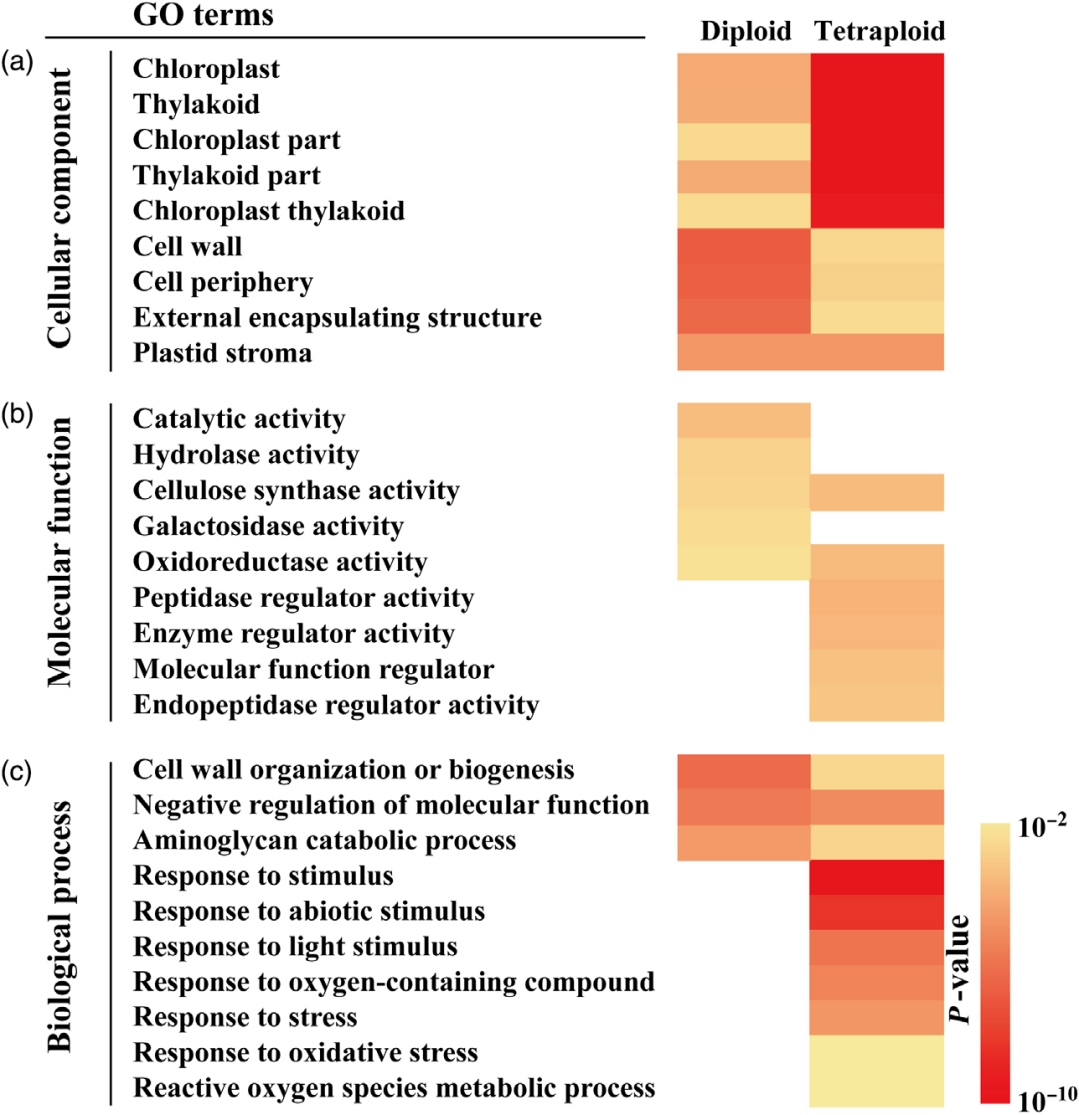
Comparative GO (gene ontology) enrichment analysis of enriched differentially expressed genes (DEGs). (a–c) Cellular component (a), molecular function (b) and biological process (c) of the DEGs in the diploid and tetraploid based on pairwise comparisons of 2D vs. 2C (diploid) and 4D vs. 4C (tetraploid) respectively. Colour panels display the *P*‐values of each GO term from 10^−10^ to 10^−2^.

The identified DEGs in 2D vs. 2C and 4D vs. 4C groups were annotated based on three major categories, cellular component, molecular function and biological process, which may reveal differences in drought stress response between the tetraploid and diploid. As is shown in Figure 4a, the top nine GO terms in the ‘cellular component’ category were similarly enriched in the tetraploid and diploid plants. As for the ‘molecular function’ category, the enriched GO terms in the diploids varied greatly from those of the tetraploids, as they only shared two GO terms, ‘cellulose synthase activity’ and ‘oxidoreductase activity’ and had few specific ones. Four GO terms, ‘peptidase regulator activity’, ‘enzyme regulator activity’, ‘molecular function regulator’ and ‘endopeptidase regulator activity’, were only enriched in the tetraploid, whereas catalytic activity, hydrolase activity and galactosidase activity were enriched the diploid (Figure 4b). Interestingly, prominent difference in the enriched GO terms was observed in the ‘biological process’ category, as no GO terms were specifically enriched in the diploid. The diploid and tetraploid shared three GO terms, whereas seven GO terms related to stress/stimulus response and antioxidant homeostasis were uniquely enriched in the tetraploid (Figure 4c).

**Figure 5:**
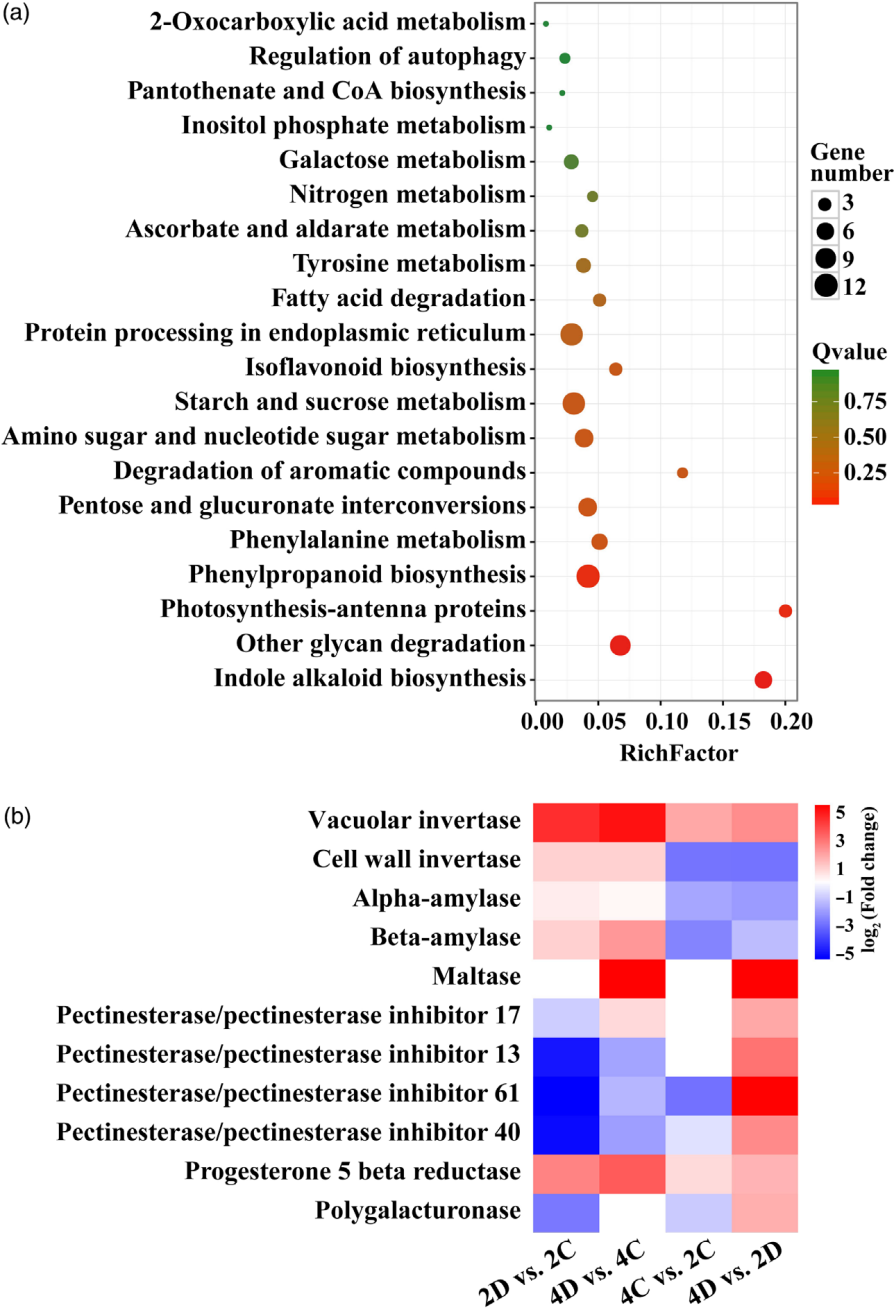
KEGG pathway analysis of the differentially expressed genes (DEGs). (a) The top 20 enriched pathways of the DEGs in the tetraploid in comparison with the diploid under drought conditions (4D vs. 2D). The *X*‐axis indicates the enrichment factor on a scale from 0 to 0.2. The dot colour and size indicate the *Q*‐value and gene number as shown on the right. (b) Heatmap of 11 genes in the diploid and tetraploid under control and drought conditions. Gradient colour barcode at the right top indicates log_2_(Fold change) values with up‐regulated genes represented by positive values and down‐regulated genes represented by negative values.

It is known that numerous genes interact with each other and function within complex pathways to play roles in abiotic stress response (Zhu, 2016). Therefore, KEGG pathway analysis of the DEGs acquired in 4D vs. 2D pairwise comparison was conducted, leading to generation of top 20 major pathways. Of note, the greatest number of enriched genes was observed in three pathways, ‘Starch and sucrose metabolism’, ‘Protein processing in endoplasmic reticulum’ and ‘Phenylpropanoid biosynthesis’ (Figure 5a). As sucrose metabolism has been previously reported to be involved in drought stress (Ruan, 2014), the 11 DEGs enriched in ‘Starch and sucrose metabolism’ pathway were further investigated (Figure 5b). We found several genes related to sugar metabolism, such as α*/*β*‐amylase*,* maltase*,* pectinesterase inhibitor* and *polygalacturonase*, were differentially expressed in the tetraploid. In particular, a vacuolar invertase gene was drastically up‐regulated by drought in both diploid and tetraploid, but the induction in the latter genotype was significantly greater (Figure 5b).

### Tetraploids accumulate lower levels of reactive oxygen species (ROS) under drought

**Figure 6:**
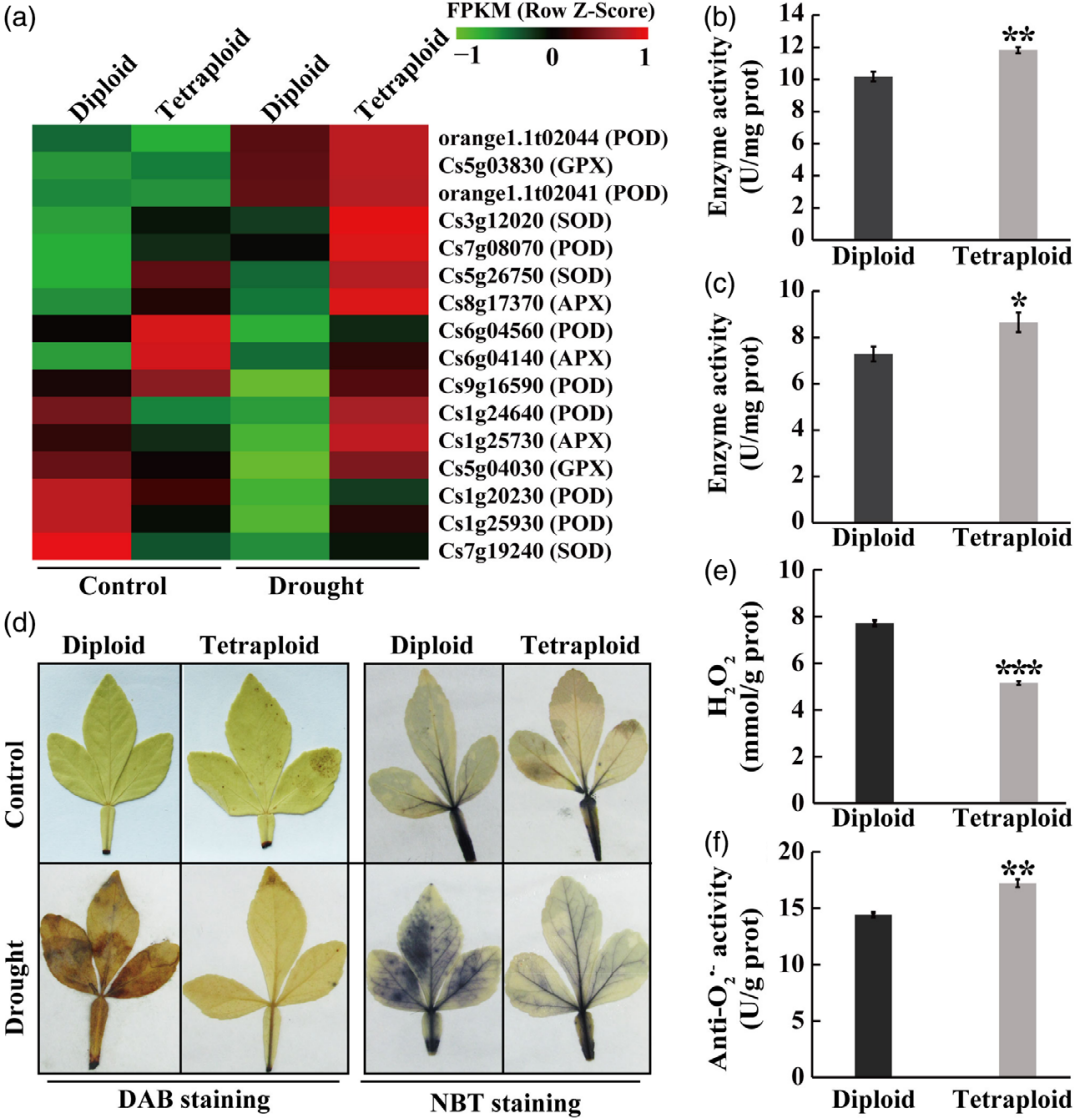
Comparison of the antioxidant system in diploid and tetraploid in response to the drought stress. (a) Heatmap showing expression levels, based on relative FPKM values, of 16 antioxidant enzyme‐encoding genes in the diploid and tetraploids under control (left two columns) and drought conditions (right two columns) conditions. The homologous gene ID in the reference genome database and corresponding enzymes are shown on the right. The gradient colour barcode at the right top indicates normalized FPKM values. (b and c) Activities of POD (b) and SOD (c) in the diploid and tetraploid plants measured after a 28‐day drought stress. (d) *In situ* accumulation of H_2_O_2_ (e) and O_2_^•−^, as revealed by histochemical staining with DAB and NBT, respectively, in the diploid and tetraploid under control (the upper row) and drought conditions (the bottom row). (e and f) Quantitative measurement of H_2_O_2_ (e) and O_2_^•−^ (f, indicated by anti‐ O_2_^•−^ capacity, which is negatively proportional to O_2_^•−^ level) in the diploid and tetraploids, measured after the drought stress. Error bars indicate SE (*n* = 3). Asterisks indicate significant differences between the diploid and tetraploid (* *P *<* *0.05; ** *P *<* *0.01; *** *P *<* *0.001).

The GO analysis indicated that four enriched GO terms in the biological process were related to antioxidant processes, implying that the diploid and tetraploid may differ in antioxidant capacity in response to drought stress. To confirm this assumption, we first analysed the expression patterns of 16 antioxidant genes enriched in one GO term, including eight *PODs*, three *SOD*s, three *APX*s and two *GPX*s. The transcript levels of all the examined antioxidant genes were distinctly higher in the tetraploid than in the diploid plants in the presence of drought (Figure 6a). Subsequently, enzyme activities of POD and SOD were measured, and results indicated that the activities of two enzymes were significantly higher in the tetraploid than in the diploid subjected to drought stress (Figure 6b, c). It is well‐known that POD and SOD are two crucial antioxidant enzymes responsible for ROS scavenging (Choudhury *et al*., 2017), so the levels of two major ROS, H_2_O_2_ and O_2_^•−^, in the diploid and tetraploid plants were examined based on histochemical staining using DAB and NBT respectively. Under non‐stressed conditions, only a slight staining was observed in the leaves of both genotypes, without any evident major differences between them. Upon exposure to the drought stress, however, the diploid displayed a deeper and more extensive staining by both DAB and NBT relative to the tetraploid (Figure 6d). *In situ* accumulation of ROS in response to the drought stress was further supported by quantitative measurements of H_2_O_2_ and O_2_^•−^ (negatively proportional to anti‐O_2_^•−^ capacity), in which the tetraploid displayed relatively lower H_2_O_2_ levels and greater anti‐O_2_^•−^ capacity than the diploid (Figure 6e, f). These results indicate that the tetraploid accumulated lower levels of ROS than the diploid in response to the drought stress.

### Tetraploids contain higher levels of glucose

**Figure 7:**
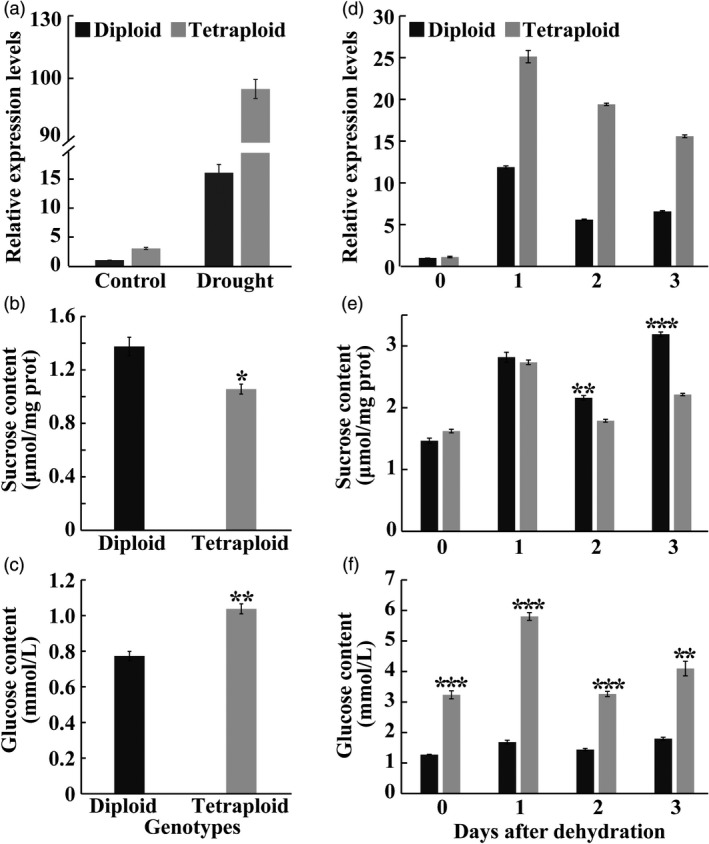
*VINV* expression and sugar content in the diploid and tetraploids in response to drought stress. (a) Transcript levels of *VINV* in the diploid and tetraploid under control and drought conditions. The expression level of diploid under control conditions is set at 1. (b and c) Contents of sucrose (b) and glucose (c) in the diploid and tetraploid leaves of trifoliate orange measured after a 28‐day drought treatment. (d–f) Transcript levels of *VINV* (d) and contents of sucrose (e) and glucose (f) in the diploid and tetraploids in response to a 3‐day dehydration treatment. Data are means ± SE (*n* = 3). Asterisks indicate significant differences between the diploid and tetraploid at the same time point (* *P *<* *0.05; ** *P *<* *0.01; *** *P *<* *0.001).

As mentioned above, the vacuolar invertase gene (*VINV*) was induced to the greater extent in the tetraploid than in the diploid. Interestingly, *VINV* was one of the four genes that was commonly up‐regulated in all four pairwise comparisons (Figure 3b), implying that it may be critical for the enhanced drought tolerance of the tetraploid. To address whether this is true, the expression level of *VINV* in diploid and tetraploid plants was examined by RT‐qPCR. Without stress conditions, *VINV* mRNA abundance was slightly higher in the tetraploid than in the diploid. Drought treatment induced a substantial up‐regulation of *VINV* in both genotypes, however, the *VINV* transcript level in the tetraploid was nearly sixfold higher than in the diploid (Figure 7a). Since vacuolar invertase functions to catalyse the irreversible conversion of sucrose into glucose and fructose (Ruan, 2014), levels of sucrose, fructose and glucose in both genotypes were measured when plants were subjected to a drought stress. Results indicated that sucrose content was significantly lower in the tetraploid, concurrent with significant elevation of glucose level (Figure 7b‐c), relative to the diploid, whereas fructose level showed no marked difference between each other (Figure S2).

An analysis of *VINV* expression and sugar levels using detached leaves subjected to a 3‐day dehydration treatment was also conducted. As with the drought stress, *VINV* was prominently up‐regulated in both genotypes by dehydration, but the expression levels in the tetraploid were significantly higher than in the diploid during dehydration process (Figure 7d). No difference in sucrose levels was detected within 1 h of dehydration, whereas the tetraploid genotype had significantly lower levels of sucrose relative to the diploid at 2 and 3 h of dehydration (Figure 7e). However, glucose levels were drastically higher in the tetraploid during the whole course of dehydration in comparison with the diploid (Figure 7f).

## Discussion

Polyploids, as an exceptional genetic and breeding resource, have been reported in recent years to be valuable for their elite attribute of greater stress tolerance. Despite the fact that many studies have been initiated to examine and characterize the stress tolerance of polyploids, advances in our understanding of the underlying mechanisms have been limited. It is conceivable that elucidation of the physiological and molecular mechanisms associated with the enhanced stress tolerance of the polyploids has vital theoretic and practical significance.

In this study, 22 trifoliate orange autotetraploids were obtained by screening a natural seedling population derived from their diploid progenitor. Polyploids are typically created artificially by using a chemical agent, like colchicine, that induces chromosome doubling; this approach is effective and has been reported to induce polyploidy in various plant species (Chen *et al*., 2016; Zeng *et al*., 2006). However, it represents a time‐consuming process and often produces chimeras (Zhang *et al*., 2007). Somatic hybridization is also a technique that can be used to obtain polyploids, but requires a high level of expertise and is very inefficient and restricted by regeneration capacity of protoplasts (Dambier *et al*., 2011). Many species of citrus and its related genera are polyembryonic and variations in genetic content (including polyploidization) often occur in asexual embryos originating from nucellar cells (Wang *et al*., 2017). Therefore, seedlings derived from the naturally derived embryo can be used to identify potential polyploids. As a matter of fact, tetraploids of citrus and trifoliate orange have been successfully obtained using this method (Aleza *et al*., 2011; Tan *et al*., 2015). Our results confirmed that this is an efficient approach for exploring tetraploids. Moreover, the tetraploids obtained by screening the seedlings were all confirmed to be autotetraploids based on genome‐wide SNP analysis. It implied that they are derived from spontaneous genome duplication of diploid trifoliate orange. Although the exact reasons for occurrence of natural polyploidization remain elusive, generation of true‐to‐type autotetraploids of trifoliate orange is reasonable as this plant is parthenocarpic.

In this study, we demonstrated that the tetraploids were more tolerant to drought and dehydration stress in comparison with their diploid progenitors based on morphological and physiological measurements. So far, tetraploids of many other plant species have been increasingly reported to exhibit enhanced tolerance to various stresses, including salt, heat and drought (Allario *et al*., 2013; Ruiz *et al*., 2016b; Zhang *et al*., 2010), implying that the tetraploids hold great potential for rendering improved stress tolerance. Trifoliate orange is a widely used rootstock for citrus grafting because of its elite traits, but it is not drought tolerant. Elevation of drought tolerance in the trifoliate orange tetraploids suggest that it may serve as a drought‐tolerant rootstock source if it meets the needs of a rootstock, which needs to be further examined when they grow to optimal size. It is worth mentioning that long‐term drought by withholding irrigation and short‐term dehydration shock were used for examining performance of the tested genotypes. As these conditions may vary from the actual situations in the nature, tolerance of the tetraploids to milder water stress conditions might be investigated in the future experiments.

The tetraploids exhibit dramatically morphological changes when compared with the diploids, which is consistent with earlier studies (Dudits *et al*., 2016; Tan *et al*., 2015). One of the major changes in the tetraploids is the smaller number of stomatal density relative to the diploids, implying that polyploidization may negatively regulate the stomatal development under normal conditions. Interestingly, the decrease in stomata density was in accordance with the enhanced dehydration and drought tolerance of the tetraploids, implying that they may lose less water via stomata‐mediated evaporation in comparison with the diploids. In addition, the leaves of tetraploids are larger and thicker than those of the diploids, which may act as another morphological factor for influencing drought tolerance. The leaves of tetraploids may allow them to confer more superior capacity for water storage and retention under the drought stress. Therefore, the leaves of tetraploids with unique anatomical and morphological changes may act as the first line of defence by protecting the plants from losing excessive water, thus contributing to the improved drought and dehydration tolerance from morphological aspects.

It is well‐known that transcriptional changes of gene expression play pivotal roles in modulation of the physiological and biological processes, including abiotic stress response (Zhu, 2016). To advance our understanding of the molecular mechanisms responsible for the improved drought tolerance in the tetraploids, we performed a comparative transcriptional analysis between diploid and tetraploids. Interestingly, the diploid had a greater number of DEGs than the tetraploid upon exposure to the drought stress. This result is similar to another study on citrus carried out by Allario *et al*. (2013), suggesting that the tetraploid plants may exhibit slight alterations in gene expression to exhibit the improved drought tolerance phenotype. Another explanation for this phenomenon is that the tetraploid plants may utilize an avoidance mechanism to combat with the drought stress, as avoidance mechanisms do not involve tremendous changes in the expression of many genes to exhibit stress resistance (Verslues *et al*., 2006). It is also interesting to note that even though the diploid genotype exhibited a greater response to drought stress, most of the DEGs were down‐regulated in the 2D vs. 2C pairwise comparison, whereas the majority of DEGs in the tetraploid genotype were up‐regulated (4D vs. 4C). Thus, these data indicate that the tetraploid genotype had relatively greater number of up‐regulated genes in response to the drought treatment than the diploid genotype. More interestingly, GO analysis on the basis of biological process indicated that the tetraploid exhibited special and extensive enrichment of genes involved in response to environmental stimulus or stresses. Our results agree well with an earlier study by Tan *et al*. (2015) who reported that the DEGs in the tetraploid were highly related to stress response. These data seem to support that genome duplication may allow the tetraploid to efficiently regulate expression of stress‐responsive genes in the presence of a stress factor. Although some genes of unknown functions were observed in these GO terms, a range of genes that have been previously shown to be implicated in stress tolerance are induced to greater extent in the tetraploids, including *RHA2b*,* CIPK5*,* PIP1*,* SRP* and *EXP1*, among others. *RHA2b* is a RING‐H2 E3 ligase that has been shown to influence drought response by regulating stomatal closure (Li *et al*., 2011). CIPKs are Calcineurin B‐like protein (CBLs)‐interacting protein kinases that function as Ca^2+^ sensors and play critical role in response to various external stimuli, such as drought (Kolukisaoglu *et al*., 2004). Plasma membrane intrinsic proteins (PIPs), belonging to aquaporin subfamily, act as water channels and play an important role in drought response (Alexandersson *et al*., 2005). SRPs encode stress‐related proteins that have been reported to exert a positive influence on drought tolerance (Kim *et al*., 2016). EXPs (Expansin), with proposed functions in cell division and elongation, are also reported to play a role in abiotic stress response (Guo *et al*., 2011). These stress‐responsive genes may play a functional role in the improved drought tolerance of the tetraploid genotype. Collectively, transcriptome reprogramming of a vast number of genes associated with abiotic stress response may constitute an important molecular mechanism pertinent to the elevated drought tolerance in the tetraploids. However, a different observation from this study was reported on the transcriptome of *Paulownia* (*P. tomentosa* × *P. fortunei*) tetraploid, in which the tetraploid exhibited a greater response to drought stress, with more down‐regulated DEGs than up‐regulated genes relative to the diploid genotype (Xu *et al*., 2014). This suggests that stress‐response mechanisms of tetraploids may vary in different plant species, and the reason for these disparities is needed to be further ascertained.

Reactive oxygen species are highly reactive, toxic molecules generated in cells; excess generation of ROS results in oxidative stress and detrimental damages to nucleic acids, proteins and lipids (Choudhury *et al*., 2017; Gill and Tuteja, 2010). Many studies have demonstrated that abiotic stresses can induce drastic accumulation of ROS, which may have profound influence on cell viability and integrity if they are not timely scavenged (Xia *et al*., 2015; Zhang *et al*., 2015b). It is conceivable that *in situ* and real‐time levels of ROS may be negatively proportional to the magnitude of stress tolerance, implying that stress tolerant genotypes may generate and accumulate less ROS in the presence of a stressor. Plants have evolved a multifaceted antioxidant defence system, involving both enzymatic and non‐enzymatic components, to prevent ROS damage when they are challenged by stressful cues (Huang *et al*., 2013; Zhu, 2016). SOD and POD are two of the most antioxidant enzymes and play a vital role in elimination of O_2_^•−^ and H_2_O_2_ respectively. Our study indicated that the tetraploid genotype exhibited higher expression levels of *SOD* and *POD* genes than the diploid in response to drought stress. In agreement with the higher transcript levels of the genes, POD and SOD activities were also prominently greater in the tetraploid than in the diploid, implying that the tetraploid may exhibit greater capacity of ROS scavenging. This is further corroborated by the less accumulation of both O_2_^•−^ and H_2_O_2_ in the tetraploid when compared with the diploid. These data demonstrate that the improved ability to prevent ROS accumulation might contribute, at least in part, to the enhanced drought stress tolerance of the tetraploid genotype. Our finding agrees with earlier studies in which autotetraploids exhibiting increased stress tolerance also displayed enhanced antioxidant system (Liu *et al*., 2011; Zhang *et al*., 2010). Therefore, enhanced ROS scavenging capacity in response to abiotic stresses may be a common effect of polyploidization that directly contributes to enhanced stress tolerance.

Soluble sugars, especially sucrose, glucose and fructose, function not only as an energy source but also as osmoprotectants in plants, indicating that they play an important role in various physiological and biological processes, including growth, development and stress response (Couée *et al*., 2006; Ruan, 2014; Sapeta *et al*., 2016). Sucrose is irreversibly hydrolysed by invertase (INV) into glucose and fructose (Roitsch and González, 2004; Ruan *et al*., 2010). Invertases in plants are classified into three types, cell‐wall INV, cytoplasmic INV and vacuolar INV (VINV) based on their subcellular locations (Dahro *et al*., 2016). In this study, the expression level of *VINV* was dramatically higher in the tetraploid than in the diploid under both non‐stressed and drought conditions. Increased levels of INV has been reported to act as a part of the stress response in a variety of plant species (Albacete *et al*., 2015; Dahro *et al*., 2016; Koonjul *et al*., 2005; Mišić *et al*., 2012). It is noted that sucrose content was reduced in the tetraploid, concomitant with an increase in glucose content, relative to the diploid, implying that the higher levels of glucose in the tetraploid might contribute to the enhanced drought tolerance. INV‐mediated hydrolysis of sucrose into hexoses has been proposed to facilitate water influx and maintain favourable osmotic adjustment under water stress conditions (Ruan *et al*., 2010). Due to this mechanism it appears that the tetraploids are perhaps able to better adapt to water‐deficit stress than diploid plants.

In summary, in this study we report identification of a number of autotetraploid trifoliate orange plants in a natural seedling population. The tetraploids exhibited extensive variations of plant phenotype and stomata development and displayed evident elevation of drought and dehydration stress tolerance in comparison with the diploid progenitor. A global transcriptome analysis using high throughput RNA‐seq identified distinct transcriptional silhouette of the DEGs in the tetraploid genotype; in particular, a number of biological GO terms associated with responses to stress/stimulus and antioxidant process were uniquely enriched in the tetraploid. We further demonstrate that the tetraploids exhibited more robust ROS scavenging capacity by elevating the expression level and activity of antioxidant enzymes than the diploid. In addition, soluble sugars were increased in the tetraploid due to enhanced *VINV* expression, leading to better buffering of osmotic stress. Therefore, it is suggested that both components (the antioxidant system and sugar metabolism) may directly contribute to the enhanced drought tolerance observed in the tetraploid plants. Information generated in this study can facilitate a better understanding of the physiological and molecular mechanisms underlying the enhanced stress tolerance observed in the polyploid plants.

## Experimental procedures

### Plant materials

Trifoliate orange fruits harvested from the trees grown in the Citrus Germplasm Repository of Huazhong Agricultural University (Wuhan, China) were used for collecting seeds, which were germinated in a greenhouse under a natural photoperiod. Tetraploid seedlings were obtained according to the methods described by Tan *et al*. (2015) and Aleza *et al*. (2011) with minor modifications. For this purpose, 2‐month‐old seedlings were morphologically screened to select potential tetraploids, where seedlings with shorter height and different leaf shape were considered as candidate tetraploids, whose ploidy levels were subsequently determined via flow cytometry (FCM) or chromosome count. The tetraploid and diploid plants were transplanted into soil pots containing a commercial soil mix (peat: perlite: vermiculite = 1 : 1 : 1), which were placed in the greenhouse and subjected to normal watering and fertilizing.

### Ploidy analysis

Flow cytometry was performed with a flow cytometer (Cyflow Space, Partec, Germany) using fresh leaves based on the manufacturer's instructions. In brief, about 0.5 cm^2^ of leaf samples were chopped in 400 μL extracting buffer (Partec HR‐A) and then stained in a solution (Partec HR‐B). After filtering, the suspension was employed for FCM, using diploid trifoliate orange as a control. Chromosome count using fresh root tips was conducted according to Dudits *et al*. (2016). Cells were stained with orcein dye and observed with an upright light microscope (Nikon 80i).

### Genetic identification of the tetraploid by SNP analysis

Genomic DNA was extracted from a randomly selected tetraploid and a diploid using the cetyltrimethyl ammonium bromide method (Wang *et al*., 2011). Genome re‐sequencing was performed in Biomarker Technologies (Beijing, China). The obtained clean reads were mapped to the pummelo (*Citrus grandis*) reference genome (http://citrus.hzau.edu.cn/orange/download/index.php) for unravelling SNPs between the diploid and tetraploid. Simultaneously, genome re‐sequencing data of three mandarin and three pummelo genotypes were downloaded from the citrus annotation project database (Xu *et al*., 2013) for identifying their SNPs by aligning to the reference genome of pummelo. A phylogenetic tree was then constructed based on the obtained SNPs from the eight genotypes using the neighbour‐joining method in MEGA5 software.

### Morphological and microscopic observation

Four‐month‐old diploid and tetraploid plants grown under the same conditions were used to assess plant height and leaf size. Stomatal density was determined by calculating stomata number on the lower leaf epidermis by examining a total of 20 visual fields using scanning electron microscopy (SEM). Longitudinal cross sections of paraffin‐embedded leaves for both diploid and tetraploid were prepared as described by Tan *et al*. (2015), and observed with a light microscope (Nikon 80i). Leaf thickness was calculated with ImageJ software (https://imagej.nih.gov/ij/).

### Stress treatments and assays

For drought stress, 4‐month‐old diploid and tetraploid seedlings were withheld irrigation for 28 day. The dehydration treatment was carried out by using detached leaves from 4‐month‐old plants and fresh shoots from 2‐year‐old plants. The detached leaves and shoots were placed on filter papers on a clean bench and kept under ambient temperature conditions for 3 or 5 day. Histochemical staining, electrolyte leakage (EL) analysis and chlorophyll fluorescence imaging were performed at the end of treatments. On the other hand, leaves were sampled at the designated time points, immediately frozen in liquid nitrogen and stored at −80 °C for further measurements of malondialdehyde (MDA), sucrose and glucose and extraction of RNA.

### Physiological measurements

Electrolyte leakage was measured as has been described by Dahro *et al*. (2016) using a conductivity meter (DSS‐307, SPSIC, China) with slight modifications. To do this, the collected leaves were immediately immersed in 15 mL of ddH_2_O and shaken for 1 h at 50 rpm. The initial conductivity values for the samples (C1) and ddH_2_O (CK1) were measured. Then, the tubes were boiled in 100 °C water bath for 10 min and then cooled down prior to measurement of the second conductivity values were measured (C2, CK2). The EL were calculated according to the formula: EL = (C1–CK1)/(C2–CK2) × 100%. For chlorophyll fluorescence detection, the leaf samples were placed in darkness for 15 min prior to imaging with an IMAGING‐PAM chlorophyll fluorometer (Walz, Effeltrich, Germany). Maximum quantum efficiency of photosystem II (*F*_v_/*F*_m_) was obtained by Imaging WinGegE software (Murchie and Lawson, 2013). Relative water loss (%) under dehydration treatment was measured by comparing leaf fresh weight at a designated timepoint with the initial one. Chlorophyll (a, b and total) content was analysed according to Dahro *et al*. (2016). Briefly, 0.1 g leaf powder was used to extract chlorophyll with 80% acetone. After centrifugation for 10 min at 7000 ***g***, the supernatants were collected and used for reading absorbance at 663, 645 and 480 nm with a spectrophotometer (Shimadzu UV‐1800, Japan). The concentrations of chlorophyll were calculated based on the absorbance and flesh weight (FW) of the samples. Histochemical staining of H_2_O_2_ and O_2_^•−^ was performed by putting the leaves in fresh solutions of 1 mg/mL 3,3′‐diaminobenzidine (DAB) or nitro blue tetrazolium (NBT) for 12 h, followed by decolorization in 80% alcohol. For analyses of MDA levels, peroxidase (POD) and superoxide dismutase (SOD) activity, H_2_O_2_, anti‐O_2_^•−^ capacity, sucrose and glucose content, 0.2 g of leaf samples were homogenized in 2 mL cold extraction buffer (0.1 m phosphate buffer, pH 7.0). After centrifugation for 10 min at 8000 rpm, the supernatants were used for measurements of the parameters using corresponding assay kits (Nanjing Jiancheng Bioengineering Institute, Nanjing, China) according to the manufacturer's instructions and as described previously (Geng and Liu, 2018; Gong *et al*., 2015). MDA, POD, SOD, H_2_O_2_, anti‐O_2_^•−^ and sucrose were calculated based on total protein content of the samples (nmol/mg protein for MDA, unit (U)/mg protein for POD and SOD, mmol/g protein for H_2_O_2_, U/g protein for anti‐O_2_^•−^, μmol/mg protein for sucrose), whereas glucose was calculated in mmol/L. Total protein content was measured using the Coomassie Brilliant Blue G‐250 staining method (Bradford, 1976).

### RNA‐seq and analysis

For transcriptome analysis, two biological replicates were used for diploid and tetraploid under well‐watered or drought conditions. To this end, total RNA was extracted using a RNAiso Plus kit (TaKaRa, Dalian, China) according to the manufacturer's instructions. RNA quality was checked by electrophoresis on 1% agarose gel and spectrophotometry with a NanoDrop™ 2000 UV‐vis Spectrophotometer (Thermo Scientific, Waltham, MA). The transcriptome analysis was performed in BGI (Shenzhen, China). Library construction was carried out according to Illumina standard instructions, and was sequenced on a BGISEQ‐500 platform. Raw reads were subjected to quality control (QC) and then filtered by removing reads with adaptors, reads with >10% uncertain base calls, low‐quality reads (containing more than 50% low‐quality bases). The resulting clean reads were aligned to the *Citrus sinensis* genome (Xu *et al*., 2013) using Bowtie2 and HISAT2 software. Fragments Per Kilobase of transcript per Million mapped reads (FPKM) method was used to calculate the expression levels of the identified genes. NOISeq software was used to identify differentially expressed genes (DEGs) between different groups as described by Tarazona *et al*. (2011). Genes with a fold change ≥2 and FDR (False Discovery Rate) < 0.01 were considered as DEGs. The DEGs were mapped to Gene Ontology (GO) terms in the database (http://www.geneontology.org/), and gene numbers for each term were calculated so as to identify significantly enriched GO terms based on a hypergeometric test. In addition, pathway enrichment analysis was conducted utilizing the KEGG database.

### Real‐time quantitative Reverse transcription PCR (RT‐qPCR) analysis

To assay the relative expression levels, RT‐qPCR analysis was performed with the total RNA extracted as described above. The total RNA was treated with RNase‐free DNase I (TaKaRa) at 42 °C for 2 min, and then used as templates (1 μg each) for first‐strand cDNA synthesis with a PrimeScript^®^ First Strand cDNA Synthesis Kit (TaKaRa, Dalian, China) according to the manufacturer's instructions. RT‐qPCR analysis with gene‐specific primers (Table S7) was conducted on a QuantStudio 7 Flex system (Applied Biosystems Foster City, CA) using SYBR Green PCR Master Mix (QIAGEN, Germany) based on the manufacturer's protocol. *ACTIN* was used as a reference gene to normalize samples, and relative gene expression levels were measured using the 2^−ΔΔCT^ method (Livak and Schmittgen, 2001). Three technical replicates were used for each sample.

### Statistical analysis

Stress treatment was repeated more than twice with consistent results, using at least three replicates for each genotype. Statistical analysis of the data, shown as means ± SE, was conducted using one‐way analysis of variance (ANOVA) and *t*‐test in SPSS (IBM, New York, NY) and Microsoft Office Excel, taking *P <* *0.05, **P <* *0.01 and ***P <* *0.001 as significance.

## Conflicts of interest

The authors declare no conflicts of interest.

## Supporting information


**Figure S1.** Genetic constitution analysis of the obtained tetraploid trifoliate oranges.
**Figure S2.** Fructose content in the diploid and tetraploids under drought (a) and dehydration (b) stresses.

**Table S1** Summary of genome re‐sequencing data of the diploid and tetraploid.
**Table S2** Summary of RNA sequencing and alignment data for each sample.
**Table S3** Differentially expressed genes in the diploid in response to drought stress relative to the normal growing conditions (2D vs. 2C).
**Table S4** Differentially expressed genes in the tetraploid in response to drought stress relative to the normal growing conditions (4D vs. 4C).
**Table S5** Differentially expressed genes in the tetraploid relative to the diploid under normal growing conditions (4C vs. 2C).
**Table S6** Differentially expressed genes in the tetraploid relative to the diploid under drought stress (4D vs. 2D).
**Table S7** List of primer sequences used in this study.
