# Supplementary material for: Enhanced ROS scavenging and sugar accumulation contribute to drought tolerance of naturally occurring autotetraploids in Poncirus trifoliata
Source: Plant Biotechnol J. 2019 Jan 10;17(7):1394–407. doi: 10.1111/pbi.13064 (PMC6576089; doi:10.1111/pbi.13064)
Supplement: Supplementary file 1 — Figure S1. Genetic constitution analysis of the obtained tetraploid trifoliate oranges. Figure S2. Fructose content in the diploid and tetraploids under drought (a) and dehydration (b) stresses. [file PBI-17-1394-s002.docx]

**Supporting Figures**


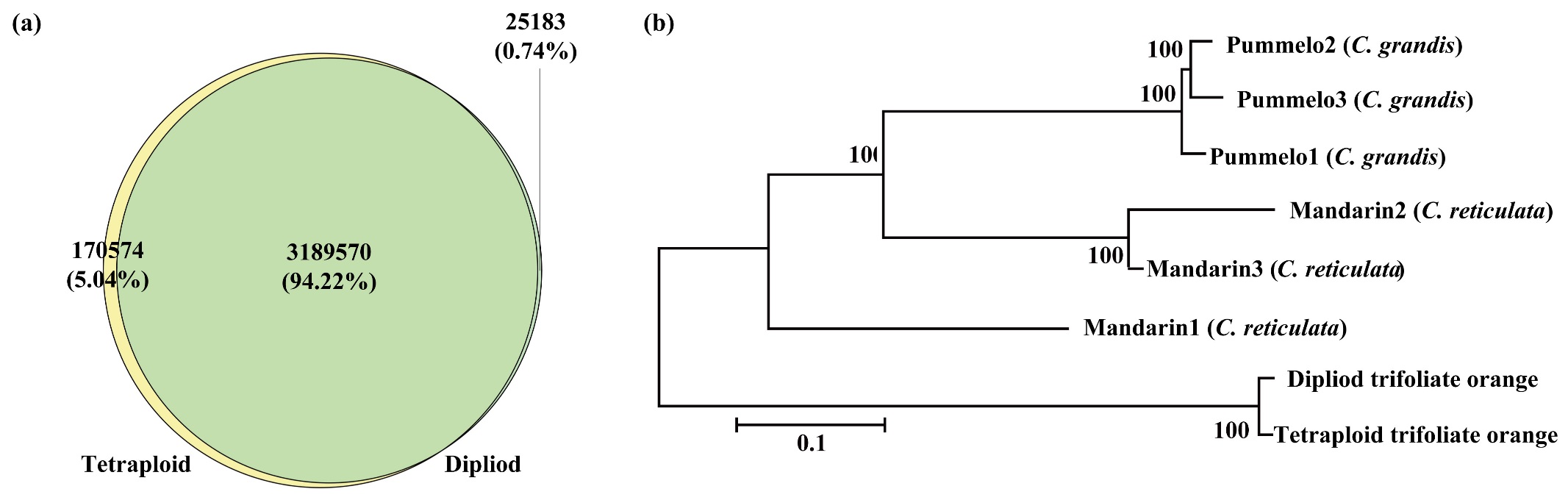


**Figure S1.** Genetic constitution analysis of the obtained tetraploid trifoliate oranges. (a) Venn diagram of SNP number and proportion between diploid and tetraploid. The numbers indicate the SNP count when aligned to the reference genome, and corresponding percentages were also indicated. (b) A phylogenetic tree was constructed using the obtained SNPs among three individual mandarins, three individual pummelos and the diploid and tetraploid trifoliate oranges. Bootstrap values = 100 are indicated.


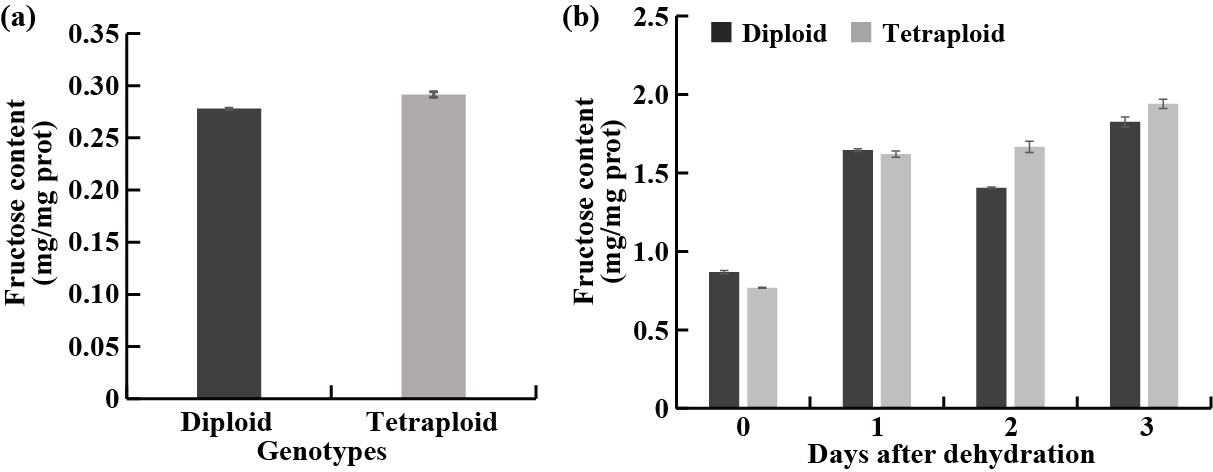


**Figure S2.** Fructose content in the diploid and tetraploid under drought (a) and dehydration (b) stresses.
